# Supplementary figures and images for: The seven transmembrane domain protein MoRgs7 functions in surface perception and undergoes coronin MoCrn1-dependent endocytosis in complex with Gα subunit MoMagA to promote cAMP signaling and appressorium formation in Magnaporthe oryzae
Source: PLoS Pathog. 2019 Feb 25;15(2):e1007382. doi: 10.1371/journal.ppat.1007382 (PMC6405168; doi:10.1371/journal.ppat.1007382)

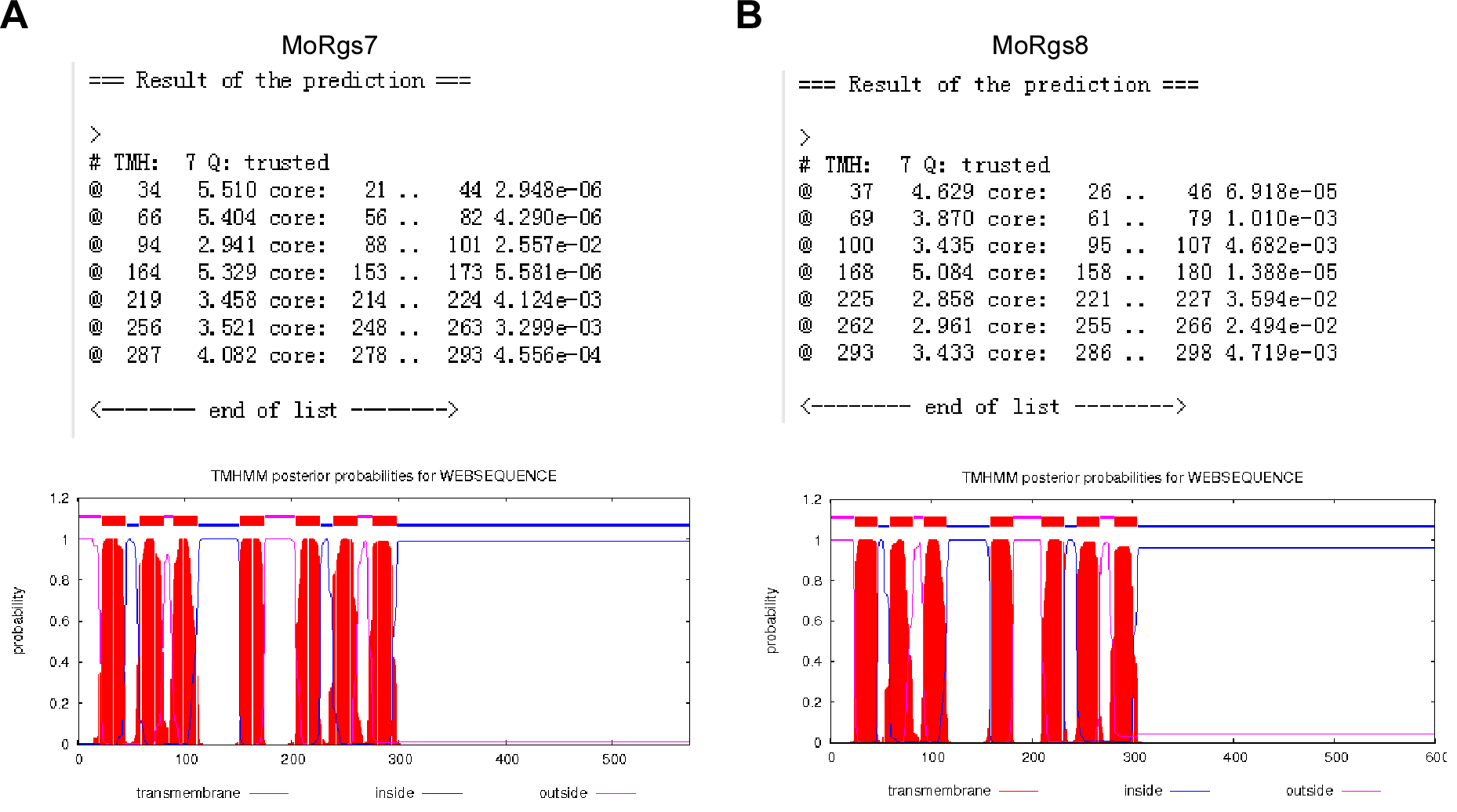

Supplement: S1 Fig — (A) The analysis results to confirm the 7-TM domain in MoRgs7 were yielded by the websites http://mendel.imp.univie.ac.at/sat/DAS/DAS.html and http://www.cbs.dtu.dk/services/TMHMM. (B) The analysis results to confirm the 7-TM domain in MoRgs8. (TIF) [file ppat.1007382.s001.tif]

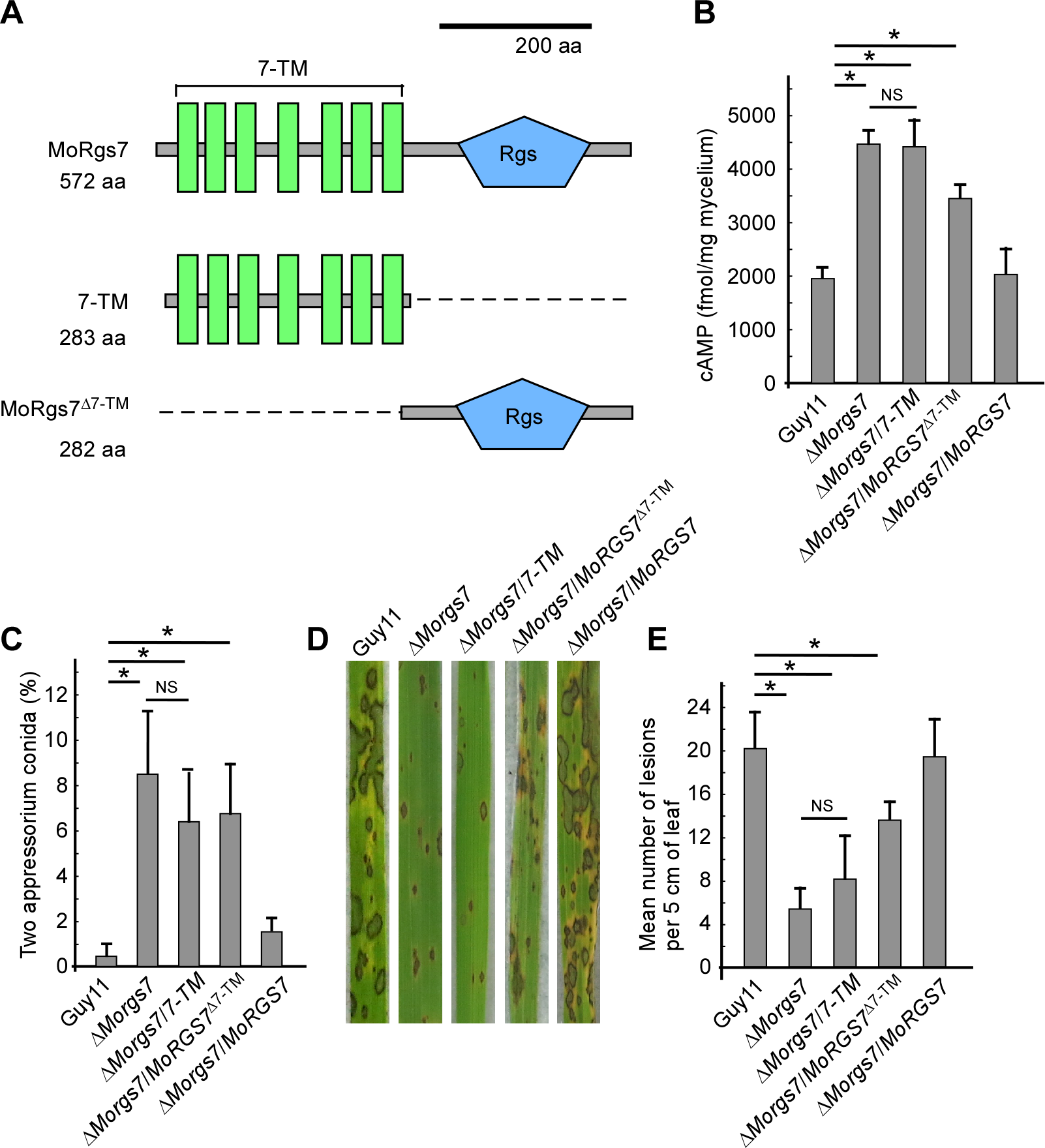

Supplement: S2 Fig — (A) The schematic representations of MoRgs7, 7-TM and MoRgs7Δ7-TM were drawn with green that represents 7-TM and blue that represents RGS domain. (B) Bar chart shows the intracellular cAMP levels in the mycelium. The values were recorded from three independent experiments. NS represents no significant differences. (C) Bar chart shows the percentages of the conidia generating two appressoria. The values were recorded from three independent experiments. NS represents no significant differences. (D) Pathogenicity assay was conducted by spaying conidial suspensions (5×10^4^ conidia/ml) onto two-week old rice seedlings (CO-39). (E) Mean number of lesions per 5 cm length of leaves were quantified for (D). (TIF) [file ppat.1007382.s002.tif]

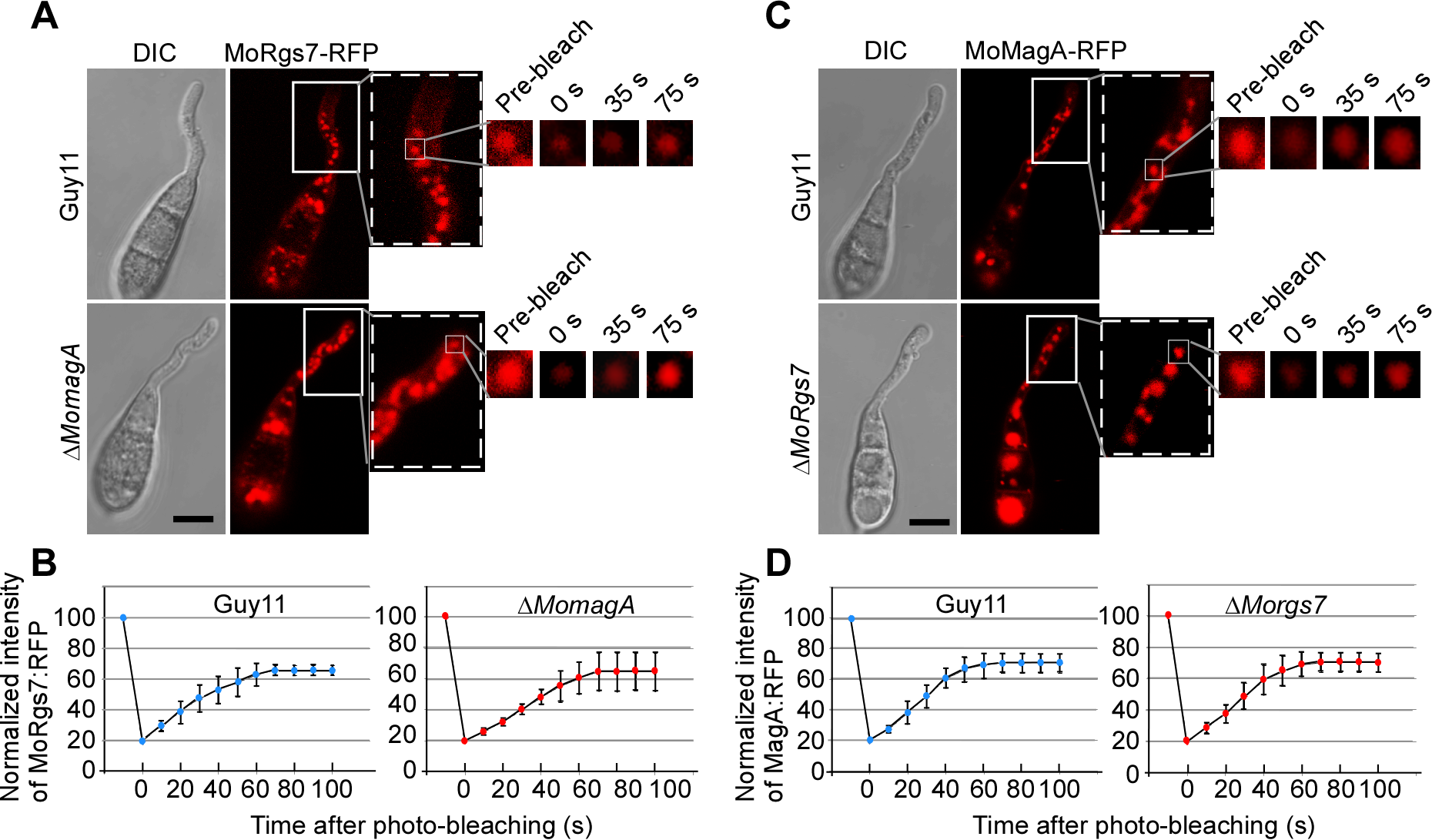

Supplement: S3 Fig — (A) The representative images of FRAP analysis for MoRgs7:RFP were shown and the selected areas were measured for fluorescence recovery after photobleaching. FRAP analysis was conducted at 3 h post-germination. Bar = 5 μm. (B) The normalized FRAP curve of MoRgs7:RFP were fitted with measuring 15 regions from different cells. (C) The representative images of FRAP analysis for MoMagA:RFP were shown and FRAP analysis was conducted at 3 h post-germination. Bar = 5 μm. (D) The normalized FRAP curve of MoMagA:RFP were fitted with measuring 15 regions from different cells. (TIF) [file ppat.1007382.s003.tif]

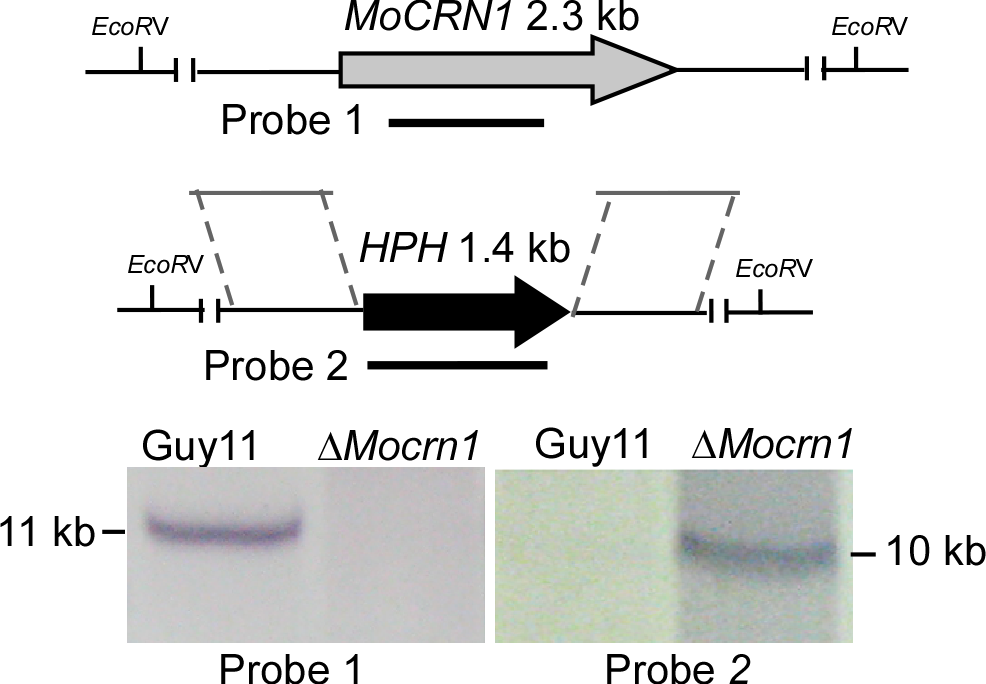

Supplement: S4 Fig — Southern blot analysis of the MoCRN1 gene deletion mutants with gene specific probe (probe1) and hygromycin phosphotransferase (HPH) probe (probe2). Thick arrows indicate the orientations of the MoCRN1and HPH genes. Thin lines below the arrows indicate sequence-specific gene probes. (TIF) [file ppat.1007382.s004.tif]

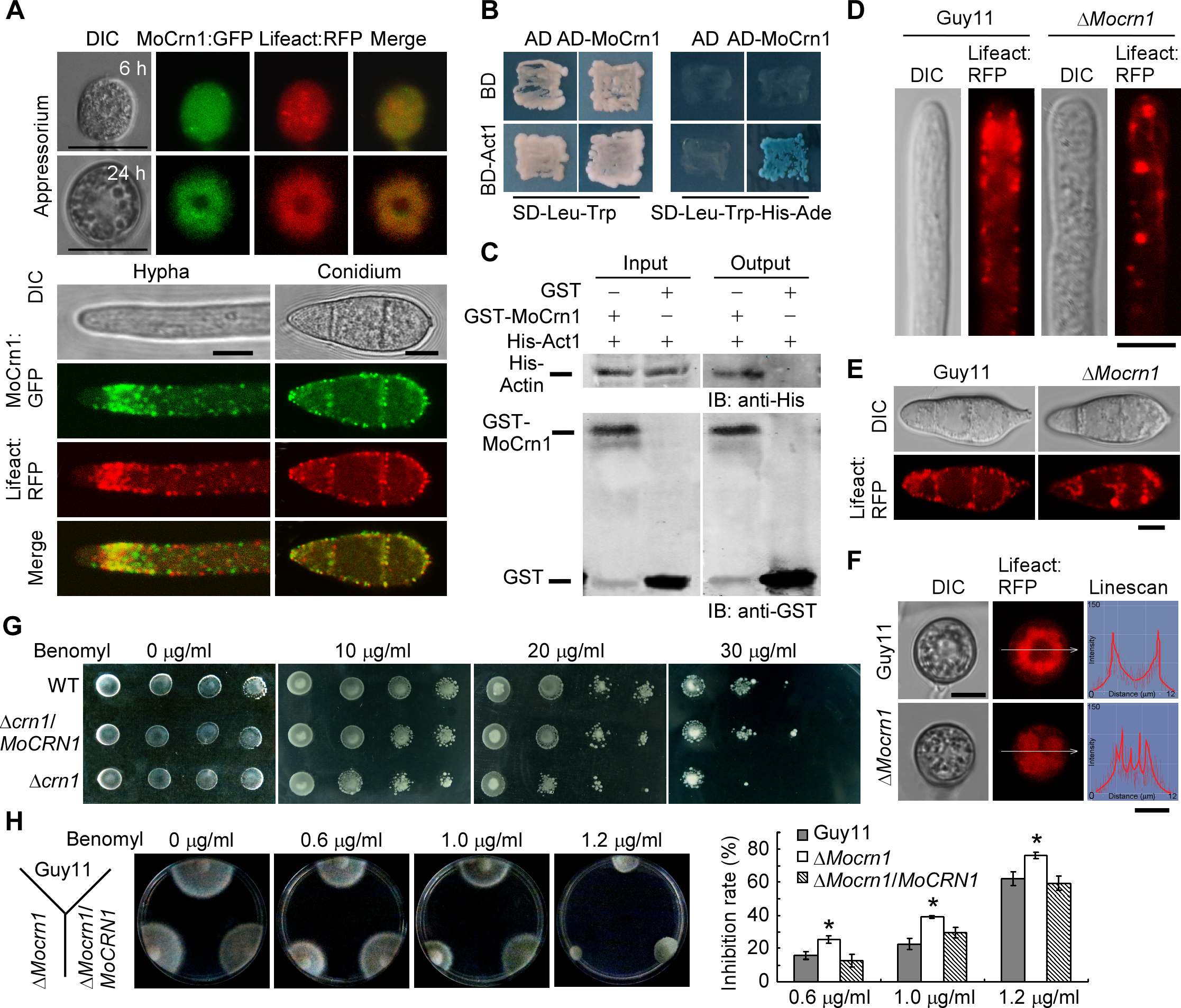

Supplement: S5 Fig — (A)MoCrn1 is co-localized with F-actin in appressorium, hypha and conidium. Scale bar for the appressorium, 10 μm. Bar of hypha, 5 μm. Bar of conidium, 5 μm. (B) The yeast two-hybrid assay for examining the interaction of MoCrn1 with actin protein MoAct1. The yeast transformants were isolated from SD-Leu-Trp plates, following growing on SD-Leu-Trp-His-Ade plates containing X-α-Gal for examining β-galactosidaseactivity. (C) Binding assay for examining the interaction of MoCrn1 with actin protein MoAct1. Input represents the proteins extracted from the E. coli BL21 strains expressing GST-MoCrn1 or His-MoAct1. Output represents the proteins eluted from the GST-beads used to bind GST-MoCrn1. Those proteins were probed by using GST-antibody and His-antibody. (D) Images show actin structures labeled by lifeact:RFP in appressoria. Bar = 5 μm. (E) Images show actin structures labeled by lifeact:RFP in conidia. Bar = 5 μm. (F) Images show actin structures labeled by lifeact:RFP in hyphae. Bar = 5 μm. (G) The assay for determining sensitivity to benomyl. The yeast wild-type BY4741, the Δcrn1 mutant and the Δcrn1Δ/MoCRN1 strains were grown on SD plates containing 0, 10, 20 and 30 μg/ml benomyl for 3 days. (H) The colonies of Guy11, the ΔMocrn1 mutant and the complemented strain grew on CM plates containing 0, 0.6, 1.0 and 1.2 μg/ml benomyl for 7 days. Bar chart shows the inhibition rate. The experiment was repeated three times. (TIF) [file ppat.1007382.s005.tif]

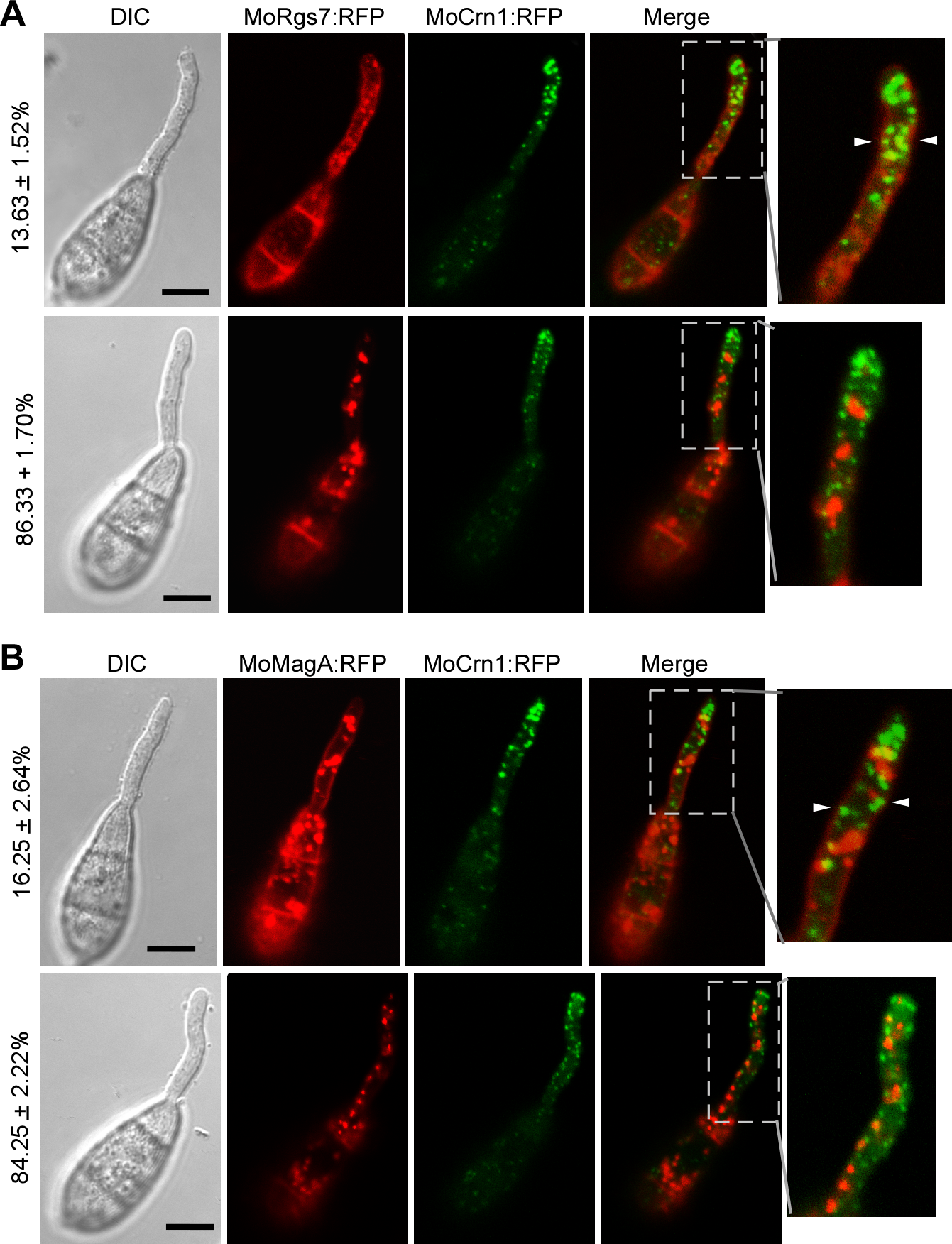

Supplement: S6 Fig — (A) The co-localization between MoCrn1:GFP and MoRgs7:RFP was examined in germ tubes at 3 h post-inoculation. The arrows indicate the sites at where MoCrn1:GFP was co-localized with PM-localized MoRgs7:RFP. Percentage of a pattern showed in image was calculated by observation for 50 germinated conidia that were randomly chosen, and observation was conducted for 3 times. Bars = 5 μm. (B) The co-localization between MoCrn1:GFP and MoMagA:RFP was examined in germ tubes at 3 h post-inoculation. The arrows indicate the sites at where MoCrn1:GFP was co-localized with PM-localized MoMagA:RFP. Percentage of a pattern showed in image was calculated by observation for 50 germinated conidia that were randomly chosen, and observation was conducted for 3 times. Bars = 5 μm. (TIF) [file ppat.1007382.s006.tif]

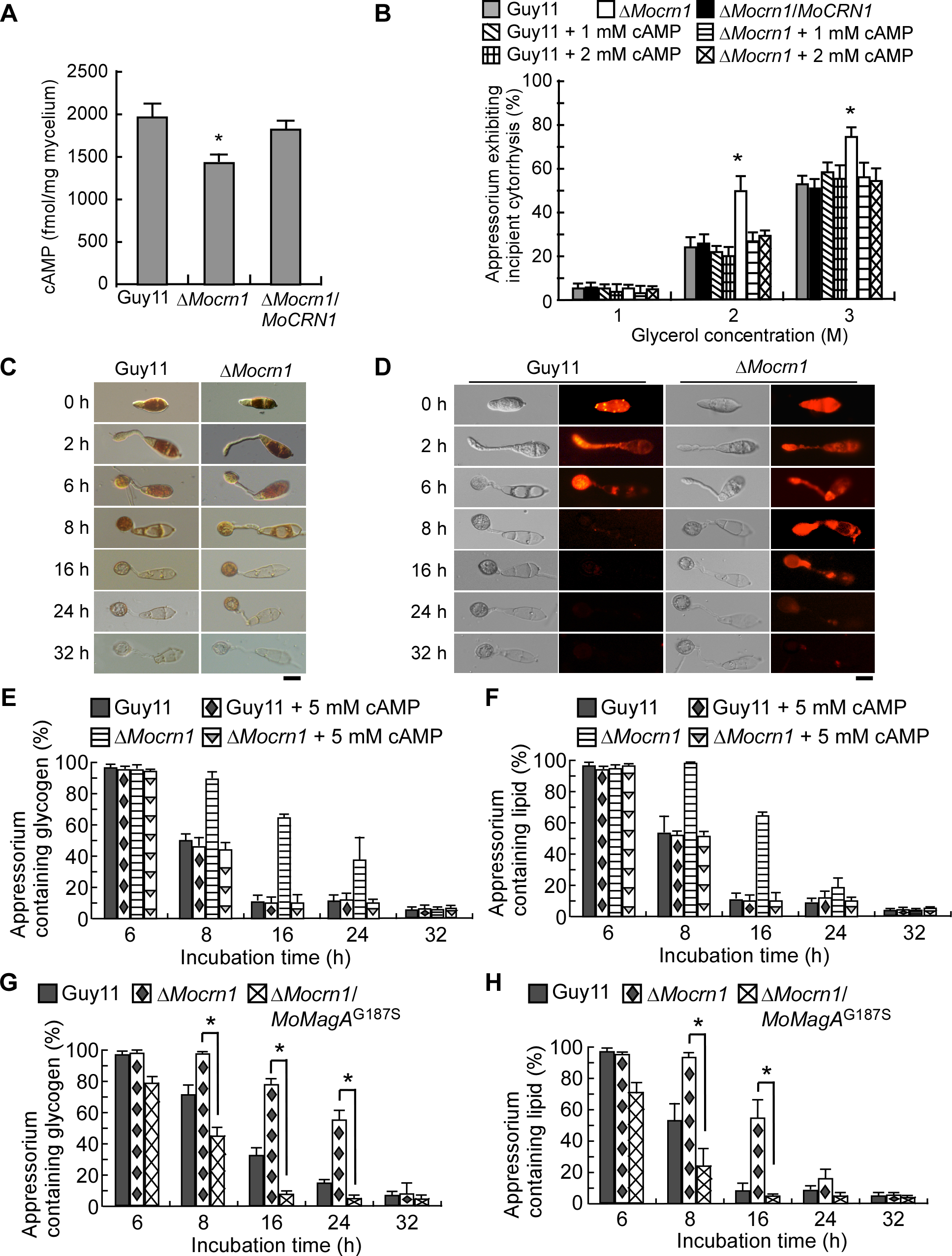

Supplement: S7 Fig — (A) Bar chart shows the intracellular cAMP levels in mycelium of Guy11, the ΔMocrn1 mutant and the complemented strain. Asterisks represent significant differences (P < 0.01). (B) Incipient collapse assay was conducted with 1, 2 and 3 M glycerol solution to examine the appressorial turgor level. Bar chart shows the percentages of collapse appressoria upon glycerol solution treatment and 8-Br-cAMP addition decreased the collapse rate of ΔMocrn1 appressoria. 200 appressoria were observed for each sample and the experiment was repeated three times. (C) Micrographs show the glycogen distribution in Guy11 and ΔMocrn1 at different time points. The conidia of Guy11 and ΔMocrn1 were allowed to germinate on hydrophobic surface, and glycogen could be visualized by iodine solution staining. Bar = 10 μm. (D) Micrographs show the lipid distribution in Guy11 and ΔMocrn1 at different time points. Lipid bodies were visualized by Nile red staining. Bar = 10 μm. (E and F) Bar charts show the percentages of appressoria containing glycogen and lipids at different time points. The 5 mM cAMP treatment significantly promoted degradation of glycogen and lipids in ΔMocrn1 appressoria. 200 appressoria were observed for each sample and the experiment was repeated three times. (G and H) Bar charts show the percentages of appressoria containing glycogen and lipids at different time points. Expressing MoMagAG187S in ΔMocrn1 significantly promoted degradation of glycogen and lipid in appressoria. 200 appressoria were observed for each sample and the experiment was repeated three times. (TIF) [file ppat.1007382.s007.tif]

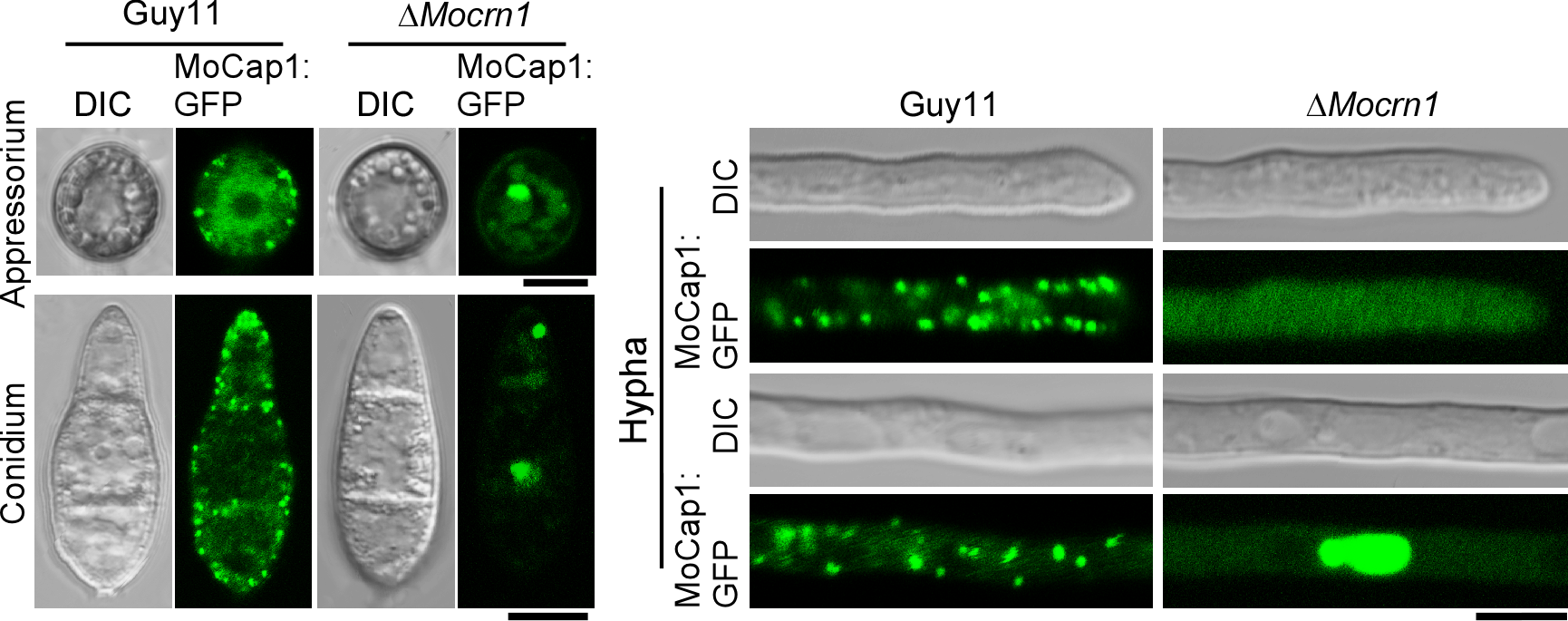

Supplement: S8 Fig — The localization pattern of MoCap1 was severely disrupted in mature appressorium, conidium and hypha of ΔMocrn1. Bars = 5 μm. (TIF) [file ppat.1007382.s008.tif]

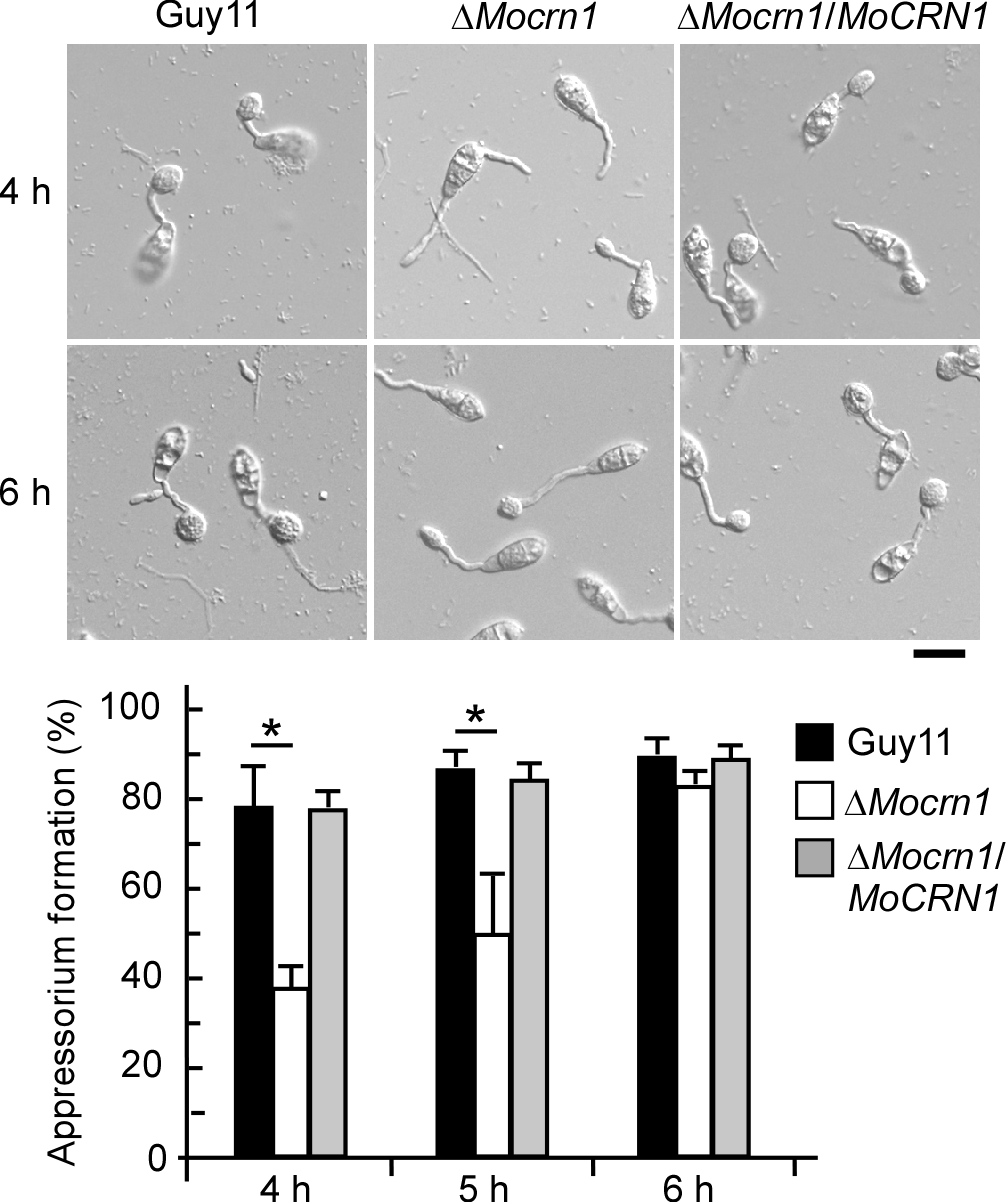

Supplement: S9 Fig — Appressoria that formed in hydrophobic surfaces were observed at 4, 5 and 6 h post-germination. Bar = 15 μm. The formation percentages were quantified by observing 200 appressoria for each sample and the experiment was repeated three times. (TIF) [file ppat.1007382.s009.tif]
