# Supplementary material for: The seven transmembrane domain protein MoRgs7 functions in surface perception and undergoes coronin MoCrn1-dependent endocytosis in complex with Gα subunit MoMagA to promote cAMP signaling and appressorium formation in Magnaporthe oryzae
Source: PLoS Pathog. 2019 Feb 25;15(2):e1007382. doi: 10.1371/journal.ppat.1007382 (PMC6405168; doi:10.1371/journal.ppat.1007382)
Supplement: S1 Table — (DOCX) [file ppat.1007382.s010.docx]

**S1 Table. Primers used in this study.**

| **Primer** | **Sequence (5’-3’)** | **Application** |
| --- | --- | --- |
| MoCRN1-Flank 1F | GTCGACTTGCGAAGGTTGAGGCGTTC | Amplifying *MoCRN1* 5' flank sequence for gene knock out |
| MoCRN1-Flank 1R | GAATTCATACTGATCACCGTGGCGAC |  |
| MoCRN1-Flank 22 | TCTAGAGCACAGTAAATGGAGGAGCC | Amplifying *MoCRN1* 3' flank sequence for gene knock out |
| MoCRN1-Flank 2R | GAGCTCGCAACTTGGGCTTCTTGCGT |  |
| MoCRN1-probeF | CTCTCCGATGTTAGGTCAAC | Amplifying probe 1 for southern blot and transformants screen |
| MoCRN1-probeR | CTCCCGAGATGGAATCAAGT |  |
| FL1111 | GGAGGTCAACACATCAATG | Amplifying probe 2 for southern blot |
| FL1112 | CTCTATTCCTTTGCCCTCG |  |
| MoCRN1-comF | ACTCACTATAGGGCGAATTGGGTACTCAAATTGGTTTGGACGGTGAAGTTAGCACA | Generating constructs of *MoCRN1*:*GFP*, *MoCRN1*^H29D^:*GFP* and *MoCRN1*^ΔC:^*GFP* for observing protein localization and for complementary assay |
| MoCRN1-comR | CACCACCCCGGTGAACAGCTCCTCGCCCTTGCTCAC CGATCGAGCAGCTTCAAGCT |  |
| MoCRN1^H29D^-GFP-1R | GCGGCTAATGTGCAGGTTGTCGTAGCAAAACTCCTTTCTCGTAGATTTGCC |  |
| MoCRN1^H29D^-GFP-2F | GCGGCTAATGTGCAGGTTGTCGTAGCAAAACTCCTTTCTCGTAGATTTGCC |  |
| MoCRN1^ΔCC^-GFP-R | CACCACCCCGGTGAACAGCTCCTCGCCCTTGCTCAC TTCGTTGGTCAGGTGGCCAATCTT |  |
| pHZ68-MoCRN1-F | CGACTCACTATAGGGCGAATTGGGTACTCAAATTGTGGACGGTGAAGTTAGCACA | Generating construct of *MoCRN1*-*C’YFP* |
| pHZ68-MoCRN1-R | GTTCGGGATCTTGCAGGCCGGGCGCGATCGAGCAGCTTCAAGCT |  |
| MagA-RFP-F | ACTCACTATAGGGCGAATTGGGTACTCAAATTGGTTGACACAGCAATTCCCACCAG | Generating construct of *MagA*:*RFP* |
| MagA-RFP-R | GTCCTCGGAGGAGGCCATCTCGACCATGTAGTCGAGCAG |  |
| RFP-F | CTGCTCGACTACATGGTCGAGATGGCCTCCTCCGAGGAC |  |
| RFP-R | ATCTCTGCCTGTGGTCCCGAGGCGCCGGTGGAGTGGCGGC |  |
| AD-MoCRN1-F | GAATTCATGGCGGGTCGCTTCGTTCGC | Generating construct of pGADT7-*MoCRN1* |
| AD-MoCRN1-R | ATCGATCGATCGAGCAGCTTCAAGCT |  |
| GST-MoCRN1-F | GAATTCGTATGGCGGGTCGCTTCGTTCGC | Generating Construct of pGEX4T-2-*MoCRN1* for expressing GST-MoCrn1 |
| GST-MoCRN1-R | GCGGCCGCCGATCGAGCAGCTTCAAGCT |  |
| MagA^G187S^-RFP-1F | ACTCACTATAGGGCGAATTGGGTACTCAAATTGGTTGACACAGCAATTCCCACCAG | Generating construct of *MagA^G187^*^:^*RFP* |
| MagA^G187S^-RFP-1R | GCTGTCCATGTGCTGGCGTTCGAATTTAGCAGCAGCGGTTTCTTTCTCGACCATGTAGTCGAGCAG |  |
| MagA^G187S^-RFP-2F | GCTAAATTCGAACGCCAGCACATGGACAGCTCGGGACCACAGGCAGAGAT |  |
| MagA^G187S^-RFP-2R | TTCGAATTTAGCAGCAGCGGTTTCTTTTTACAGAATACCTGAGTCCTT |  |
| pYES2-MoCRN1-F | CGGGGTACCGATGGCGGGTCGCTTCGTTCG | Generating construct of pYES2-*MoCRN1* for yeast complementation |
| pYES2-MoCRN1-R | TGCTCTAGATCGATCGAGCAGCTTCAAGCT |  |
| BD-RGS7-F | TGATCTCAGAGGAGGACCTGCATATGATGGCAAATGCAACATCGAC | Generating construct of pGBKT7-*MoRGS7* |
| BD--RGS7-R | GCAGGTCGACGGATCCCCGGGAATTCTCAAAGGGCAGCCCCGCCGA |  |
| pHZ65--RGS7-F | CGACTCACTATAGGGCGAATTGGGTACTCAAATTGTGCTCAAGTGCCCGTATGTG | Generating construct of pHZ65-*MoRGS7* for expressing MoRgs7-N’YFP |
| pHZ65--RGS7-R | GCTCACCATCGTGGCGATGGAGCGAAGGGCAGCCCCGCCGAACT |  |
| pHZ65-MagA-1F | CGACTCACTATAGGGCGAATTGGGTACTCAAATTGGATCTAGGTACGGTACCTTG | Generating construct of pHZ65-*MagA* for expressing MagA:N’YFP |
| pHZ65-MagA-1R | CTCGACCATGTAGTCGAGCAGA |  |
| pHZ65-MagA-2F | AGCCACAACGTCTATATCATGTCGGGACCACAGGCAGAGAT |  |
| pHZ65-MagA-2R | GCTCACCATCGTGGCGATGGAGCG TTACAGAATACCTGAGTCCT |  |
| YFP-F | TCTGCTCGACTACATGGTCGAGATGGTGAGCAAGGGCGAGGA |  |
| YFP-R | CATGATATAGACGTTGTGGCT |  |
| Pro+R7F | ACTCACTATAGGGCGAATTGGGTACTCAAATTGGTTTGCTCAAGTGCCCGTATGTG | Generating constructs of MoRgs7:GFP, MoRgs7:RFP and MoRgs7^Δ7TM^:GFP |
| Pro+R7R | AAGGGCAGCCCCGCCGAACT |  |
| RFP-F | AGTTCGGCGGGGCTGCCCTTATGGCCTCCTCCGAGGACGT |  |
| RFP-R | CACCACCCCGGTGAACAGCTCCTCGCCCTTGCTCAC TTAGGCGCCGGTGGAGTGGC |  |
| 7TM-1F | TTTCGTAGGAACCCAATCTTCAAAATGGCAAATGCAACATCGAC | Generating construct of 7TM:GFP |
| 7TM-1R | CACCACCCCGGTGAACAGCTCCTCGCCCTTGCTCAC GGTCAGGACCTGCCAGCAAG |  |
| 7TM-2F | TTTCGTAGGAACCCAATCTTCAAAATGCACAAAACCCTGCGCCG |  |
| 7TM-2R | CACCACCCCGGTGAACAGCTCCTCGCCCTTGCTCAC AAGGGCAGCCCCGCCGAACT |  |
| MagA-S-1F | ACTCACTATAGGGCGAATTGGGTACTCAAATTGGTTGACACAGCAATTCCCACCAG | Generating constructs of pXY203*:MagA*:*S* and pXY203*:MagA*^G187S^:*S* |
| MagA-S-1R | GCTGTCCATGTGCTGGCGTTCGAATTTAGCAGCAGCGGTTTCTTTCTCGACCATGTAGTCGAGCAG |  |
| MagA-S-2F | GCTAAATTCGAACGCCAGCACATGGACAGCTCGGGACCACAGGCAGAGAT |  |
| MagA-S-2R | TTCGAATTTAGCAGCAGCGGTTTCTTTTTACAGAATACCTGAGTCCTT |  |
